# GAS6-AS1 Overexpression Increases GIMAP6 Expression and Inhibits Lung Adenocarcinoma Progression by Sponging miR-24-3p

**DOI:** 10.3389/fonc.2021.645771

**Published:** 2021-08-26

**Authors:** Yuanyong Wang, Minge Ma, Chuan Li, Yuling Yang, Maolong Wang

**Affiliations:** ^1^Department of Thoracic Surgery, The Affiliated Hospital of Qingdao University, Qingdao, China; ^2^Department of Radiology, The Affiliated Hospital of Qingdao University, Qingdao, China; ^3^Department of Infectious Diseases, The Affiliated Hospital of Qingdao University, Qingdao, China

**Keywords:** GAS6-AS1, lung adenocarcinoma, GIMAP6, miR-24-3p, ceRNA

## Abstract

GAS6 antisense RNA 1 (GAS6-AS1) is a long non-coding RNA involved in hepatocellular carcinoma and gastric cancer. However, the functional role of GAS6-AS1 in lung adenocarcinoma (LUAD) remains unclear. In the present study, qRT-PCR was used to measure the levels of GAS6-AS1, GIMAP6 and miR-24-3p expression in LUAD samples and cell lines. CCK-8 and colony formation assays were used to determine cell proliferation. Cell migration and invasion were evaluated using wound healing and transwell assays, respectively. The potential interactions between molecules were assessed using RNA immunoprecipitation and luciferase reporter assays. Western blot analysis was used to quantify protein expression. The anti-tumor effect of over-expressed GAS6-AS1 on LUAD was also examined *in vivo* in xenograft tumor experiments. The expression of GAS6-AS1 was notably downregulated in LUAD samples and cell lines and associated with a poor prognosis. GAS6-AS1 overexpression inhibited the migration and invasion of A549 and H1650 cells. Down-expressed GAS6-AS1 acted as a sponge for miR-24-3p and down-regulated the expression of its target, GTPase IMAP Family Member 6. These findings suggested that GAS6-AS1 might represent a potential diagnostic biomarker for LUAD.

## Introduction

Lung cancer has the highest incidence and mortality in China and can be divided into non-small cell lung cancer (NSCLC) and small cell lung cancer (1). NSCLC is further categorized as lung adenocarcinoma (LUAD) and lung squamous cell carcinoma (2). The incidence of lung adenocarcinoma has risen in recent years (3). With the development of imaging technology and the combined application of surgery, radiotherapy and chemotherapy, the survival rates of patients with LUAD are gradually improving. However, overall survival rates are still under 25% (4, 5). Therefore, it is essential to understand LUAD progression in order to identify potential therapeutic agents and diagnostic biomarkers.

Long non-coding RNA (lncRNA) molecules are a novel class of non-coding RNA with limited functional protein-coding ability (6). In humans, lncRNAs have been shown to be widely distributed and expressed in every organ (7). Previous studies have demonstrated that lncRNAs play a vital role in inhibiting oncogenes and preventing the occurrence of malignant tumors (8, 9). Several lncRNAs are aberrantly expressed in LUAD, leading to tumor inhibition or carcinogenicity depending on different mechanisms, including sponging and post-transcriptional regulation (10–13).

Growth arrest-specific 6 antisense RNA 1 (GAS6-AS1) is detectable in several types of malignant tumors, such as hepatocellular carcinoma. The increased expression of GAS6-AS1 was related to tumor size, edmondson grade and (TNM) stage of tumor-lymph node-metastasis. The overall survival time of HCC patients characterized by high expression of GAS6-AS1 was significantly shorter than that of patients with low expression. It was also proved that GAS6-AS1/miR-585/EIF5A2 pathway played an important role in the progression of hepatocellular carcinoma (14), Zhang et al. indicated that indicate that GAS6-AS1 significantly driving the aggressive phenotype in gastric cancer through activating its cognate sense gene GAS6 (15). A previous study suggested that GAS6-AS1 may be associated with LUAD, although the underlying mechanism is still unclear (16). Therefore, the aim of this study was to examine the function and potential mechanism of GAS6-AS1 in NSCLC.

## Materials and Methods

### Tissue Samples

In total, 74 pairs of LUAD samples and adjacent normal tissues were collected at the Affiliated Hospital of Qingdao University from patients who had not received chemotherapy or radiotherapy. This study was approved by the Ethics Committee of Affiliated Hospital of Qingdao University (QYFYWZLL-25569). All tissue samples were stored at -80°C until RNA extraction.

### Cell Culture and Transfection

LUAD cell lines (A549, H1299, H157 and H1650) and a human bronchial epithelial cell line (HBE) were cultured in DMEM with 10% FBS (Gibco; Thermo Fisher Scientific, Inc., USA). All cell lines were maintained in a humidified atmosphere at 37°C with 5% CO_2_.

GAS6-AS1 overexpression (OE-GAS6-AS1) plasmids and control vector (Vector), microRNA (miR) negative control (miR-NC) and miR-24-3p inhibitor were synthesized by BGI (Qingdao, China). The cells were inoculated into a 6-well plate (2 × 10^5^ cells/well), then transfected with Vector, OE-GAS6-AS1, miR-NC, miR-24-3p inhibitor either alone or in various combinations using the Polyplus-transfection^®^ reagent (Illkirch, France).

### Quantitative Real-Time PCR (qRT-PCR)

Total RNA was extracted from LUAD tissue samples or cells using TRIzol (Invitrogen; Thermo Fisher Scientific, Inc.). The SYBR Green PCR Kit (Takara, Dalian, China) was used for qRT-PCR analysis of lncRNA, miRNA and mRNA expression levels. Primer sequences (Tsingke, Qingdao, China) are listed in **Table 1**. GAPDH was used as an internal control for lncRNA and mRNA, whereas U6 was used for miRNA.

**Table 1:** Primers of Gene.

GAS6-AS1	F:GTGGGTACTGCATTCCTACCG
	R:CTCTCCTCTGATGGCAGGAC
GIMAP6	F:AGACGCTATCTGCCAAGCC
	R:GGCCCAGTTGTGTCACCAG
GAPDH	F:CTGACTTCAACAGCGACACC
	R:TGCTGTAGCCAAATTCGTTGT
miR-24-3p	F:TGGCTCACATCAGCAGGAACA
U6	F:ATTGGAACGATACAGAGAAGATT
	R:GGAACGCTTCACGAATTTG

### Cell Proliferation and Colony Formation Assay

Cell Counting Kit-8 (CCK-8, Dojindo Molecular Technologies, Inc., Japan) was used to assess cell proliferation. At 0, 24, 48 and 72 h following transfection, the OD value at 450 nm was measured using an enzyme-labeling instrument (Biotek Instruments, Inc., USA). Approximately 0.5-1 × 10^3^ transfected cells/well were seeded into 6-well plates for two weeks. The cells were then fixed with 75% ethanol and stained with 0.1% crystal violet. Lastly, the colonies were counted under a microscope.

### Wound Healing Assay

Transfected cells were seeded into a 6-well Petri dish and cultured to 80% confluence. A 10-μl pipette tip was then used to scratch the cell layer. Images of the wound were taken at different time points (0 and 48 h) under a microscope (Nikon Corporation, China).

### Apoptosis Assay

Briefly, two days following transfection, cell apoptosis was measured using an Annexin-V FITC Apoptosis Kit (BioLegend, Inc., USA). Determination of the percentage of apoptotic cells was carried out using flow cytometry (BD Biosciences, USA).

### Transwell Invasion Assay

A total of 5 × 10^4^ transfected cells were added to 300 μl serum-free medium with Matrigel. Complete DMEM was added to the lower chamber to detect the invasive ability of the cells using a transwell assay (Corning, Inc.). After 24-h incubation, the number of invasive cells was counted under a microscope (Nikon Corporation, China), fixed with paraformaldehyde and stained with crystal violet.

### Cell Nuclear and Cytoplasmic RNA Isolation

The isolation of subcellular RNAs in LUAD cells was performed using a PARIS Kit (Thermo Fisher Scientific, Inc.). qRT-PCR was then used to measure the expression levels of GAS6-AS1 in the nuclear or cytoplasmic fraction. GAPDH served as a cytoplasmic control, whereas U6 was used as a nuclear marker.

### Luciferase Reporter Assay

The GAS6-AS1 wild-type (Wt) and mutant (Mut) 3’-UTR covering the predicted miR-24-3p binding sequence and a Mut-GAS6-AS1 3’-UTR fragment, respectively, were amplified. The sequences were then inserted into the psiCHECK2 vector (Promega Corporation, USA) to construct the GAS6-AS1-Wt and -Mut plasmids. The GIMAP6 plasmids were generated using similar experimental steps. After two days, the cells were used for a dual luciferase reporter assay.

### RNA Immunoprecipitation Assay

The Magna RIP™ RNA-Binding Protein Immunoprecipitation Kit was used to perform a RIP assay according to the manufacturer’s instructions (Milibo, USA). Total RNA was purified and qRT-PCR was performed to detect the expression levels of GAS6-AS1 and miR-24-3p.

### Western Blot Analysis

Total proteins were lysed in RIPA buffer (Beyotime, China) and quantified with a BCA protein Assay Kit (Thermo Fisher Scientific, Inc.), according to the manufacturers’ protocol. Each protein sample (50 μg) was transferred to a polyvinylidene fluoride membrane. The membrane was blocked in 5% skimmed milk at room temperature for 1 h, and incubated with the following primary antibodies: β-actin (Abcam, MA, USA) and anti-GIMAP6 (Abcam, MA, USA). The membranes were then probed with secondary antibodies for 2 h and photographed. The protein levels of β-actin served as the control. Image J was used to analyze the gray value of western blot protein bands.

### Tumor Xenograft Experiment

Four-week-old nude mice were obtained from the Laboratory Animal Center of Qingdao University. A total of 2 × 10^7^ A549 cells transfected with OE-GAS6-AS1 or vector were subcutaneously injected into the flanks of the mice (n = 3 in each group). All processes were carried out in full accordance with the ARRIVE guidelines (17).

### Bioinformatics Analysis

The GEPIA database (http://gepia2.cancer-pku.cn/#index) was used to examine gene expression levels and survival rates using TCGA data. StarBase 3.0 (http://starbase.sysu.edu.cn/) was used to predict the binding sequence between GAS6-AS1, miR-24-3p and GIMAP6.

### Statistical Analysis

The data were analyzed with the SPSS 26.0 software (IBM Corp., USA) and GraphPad Prism (Version 7.0). Student’s t-test was used to assess the differences between two groups. One-way ANOVA was used to compare more groups. The correlation between GAS6-AS1 and miR-24-3p or GIMAP6 was analyzed using Spearman’s correlation analysis. All procedures were repeated 3 times.

## Results

### Low Expression of GAS6-AS1 Is Associated With Poor Prognosis in LUAD

**Figure 1:**
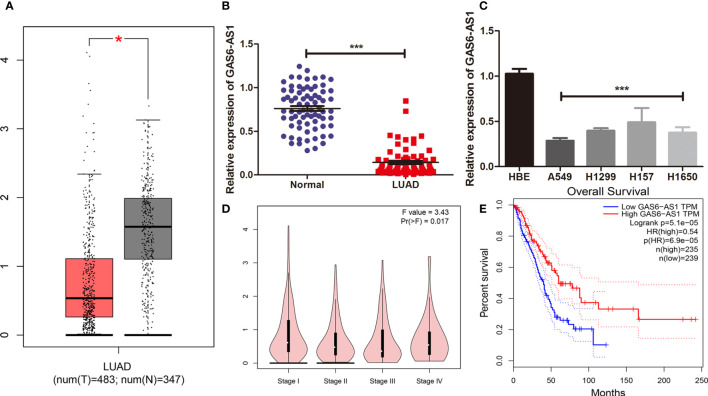
GAS6-AS1 expression levels in LUAD samples and cells. **(A)** The expression of GAS6-AS1 was analyzed using TCGA. Red represented tumor samples, white represented normal samples. **(B)** The expression of GAS6-AS1 was measured by qRT-PCR in LUAD and adjacent normal tissues (n = 74). **(C)** The expression of GAS6-AS1 was measured in LUAD cell lines and a normal bronchial epithelial cell line. **(D)** Expression levels of GAS6-AS1 according to clinical stage. **(E)** Overall survival analysis in LUAD samples according to GAS6-AS1 expression. *P < 0.05 and ***P < 0.001.

The GAS6-AS1 expression levels in LUAD were examined in a database and in tissues. As shown in **Figure 1A**, GAS6-AS1 expression was significantly downregulated in tumor samples from TCGA. qRT-PCR was also conducted to detect GAS6-AS1 mRNA expression levels in 74 pairs of LUAD tissues, which were notably decreased in the tumor group (**Figure 1B**). Furthermore, GAS6-AS1 expression was determined in four LUAD cell lines (A549, H1299, H157 and H1650) and a human bronchial epithelial cell line (HBE). GAS6-AS1 was significantly downregulated in all LUAD cell lines (**Figure 1C**).

The clinical value of GAS6-AS1 was assessed in 74 patients with LUAD who were divided into low- or high-expression groups according to the median GAS6-AS1 expression, which is the cutoff value is 4.538. As displayed in **Table 2**, decreased expression of GAS6-AS1 was significantly associated with tumor size and clinical stage, which could be used to monitor the patient’s prognosis or recurrence in the future. The online GEPIA database also verified these mentioned clinical features (**Figure 1D**). Moreover, the overall survival rate of patients with low expression of GAS6-AS1 was poor (**Figure 1E**). Therefore, GAS6-AS1 is downregulated in LUAD samples and might be associated with poor prognosis.

**Table 2:** Association of GAS6-AS1 expression with clinicopathologic factors of 74 LUAD patients.

Variables	Cases	GAS6-AS1 expression		P-value
		Low	High	
Age (years)				0.239
<60	31	13	18	
≥60	43	24	19	
Gender				0.182
Male	48	26	22	
Female	26	9	15	
Clinical stage				0.042^*^
I	52	22	30	
II-IIIa	22	15	7	
Histological grade				0.288
Well/Moderate	55	29	30	
Poor	19	12	7	
Tumor size				0.03^*^
≤1cm	46	28	19	
>1cm	28	9	18	
Lymph node metastasis				0.809
Yes	27	13	14	
No	47	24	23	

*p < 0.05.

### GAS6-AS1 Overexpression Inhibits Cell Proliferation, Migration, Invasion and Apoptosis *In Vitro*

**Figure 2:**
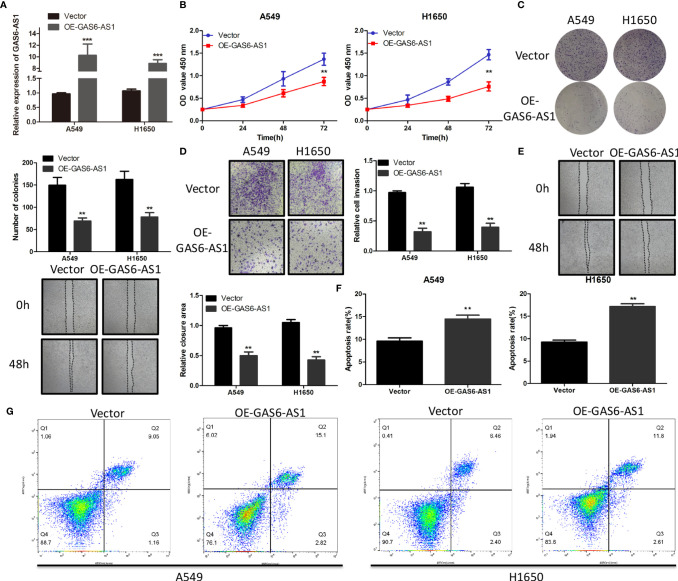
OE-GAS6-AS1 inhibits cell proliferation *in vitro*. **(A)** The level of GAS6-AS1 was measured in A549 and H1650 cells transfected with Vector or OE-GAS6-AS1. **(B, C)** Assessment of proliferation using CCK8 **(B)** and colony formation assays **(B)** in A549 and H1650 cells transfected with Vector or OE-GAS6-AS1. **(D, E)** Cell invasion and migration were evaluated using transwell **(D)** and wound-healing assays **(E)** in LUAD cells transfected with Vector or OE-GAS-AS1. **(F, G)** The effect of GAS6-AS1 upregulation on the apoptosis of A549 and H1650 cells was determined using flow cytometry. **P < 0.01 and ***P < 0.001.

A549 and H1650 cells were transfected with vector or OE-GAS6-AS1 to evaluate the effect of GAS6-AS1 in LUAD cells. The results indicated that the expression of GAS6-AS1 in cells transfected with OE-GAS6-AS1 was significantly higher than that of cells transfected with vector (**Figure 2A**). The effect of GAS6-AS1 on the proliferation of LUAD cells was assessed using a CCK-8 kit. Overexpression of GAS6-AS1 decreased the proliferation of A549 and H1650 cells (**Figure 2B**). In addition, in a colony formation assay, A549 and H1650 cells transfected with OE-GAS6-AS1 formed significantly fewer colonies than the vector group (**Figure 2C**). Similarly, transwell invasion and wound healing assays were also used to assess cell invasion and migration in transfected A549 and H1650 cells. The migration and invasion abilities of LUAD cells decreased significantly following GAS6-AS1 overexpression (**Figures 2D, E**).

Flow cytometry demonstrated that the proportion of apoptotic cells in LUAD cells overexpressing GAS6-AS1 was higher (**Figures 2F, G**). These data indicated that OE-GAS6-AS1 might promote LUAD cell death *in vitro*.

Previous studies have suggested that GAS6 may also lead to the occurrence and development of LUAD (18, 19). Thus, OE-GAS6-AS1 was also stably transfected into LUAD cells to detect the level of GAS6 expression. However, as shown in **Additional Figure 1**, the expression of GAS6 did not change with changes in GAS6-AS1 in LUAD cells.

### GAS6-AS1 Sponges miR-24-3p in LUAD Cells

**Figure 3:**
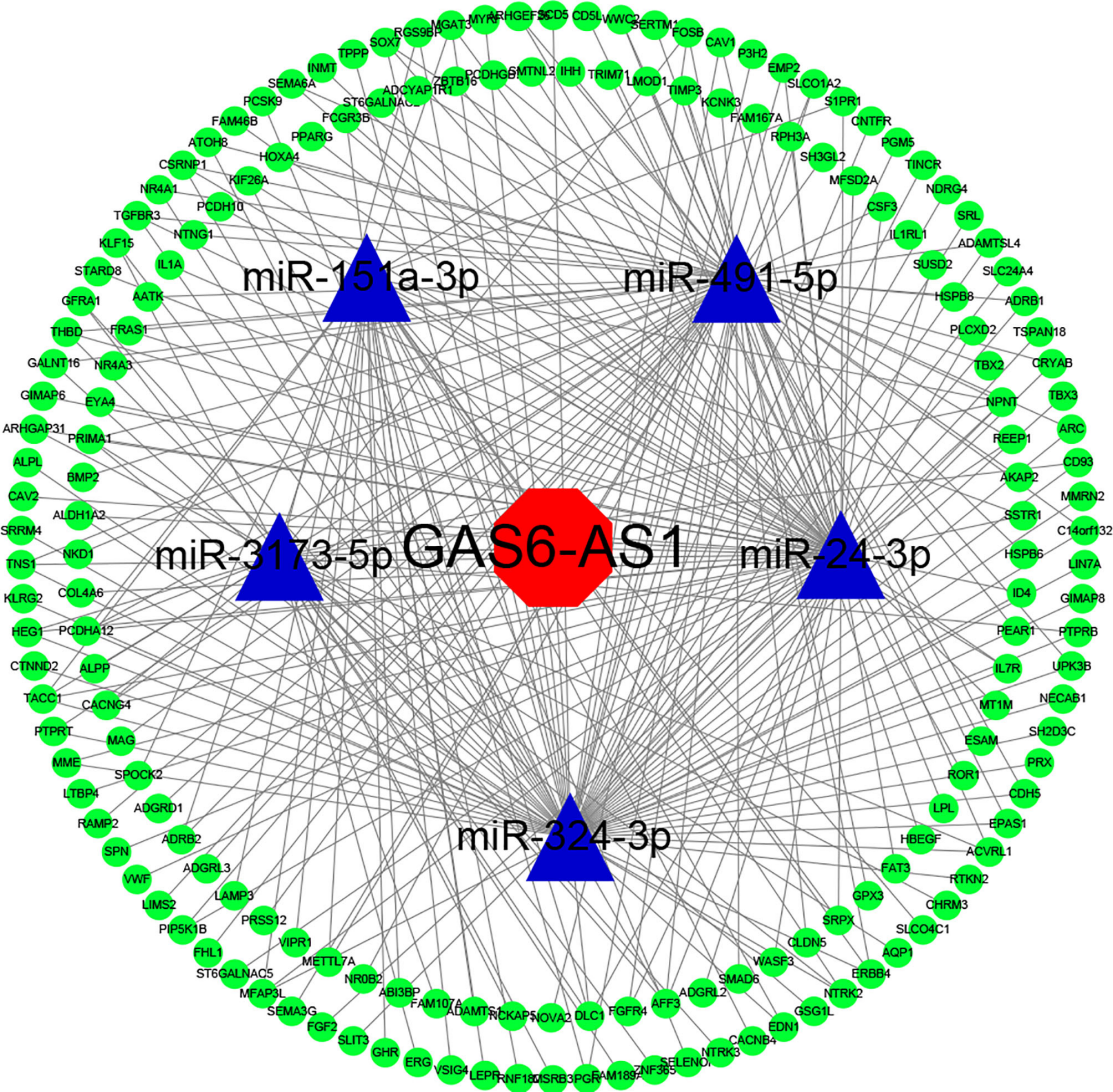
The GAS6-AS1 associated lncRNA-miRNA-mRNA ceRNA network in LUAD. StarBase was used to predict miRNAs targeted by GAS6-AS1. Both miRbase and StarBase were used to construct the predicted mRNA-miRNA and miRNA-lncRNA associations, respectively. Cytoscape (version 3.6.1) was used to construct a visual ceRNA network. Red, lncRNA. Blue, miRNA. Green, mRNA.

**Figure 4:**
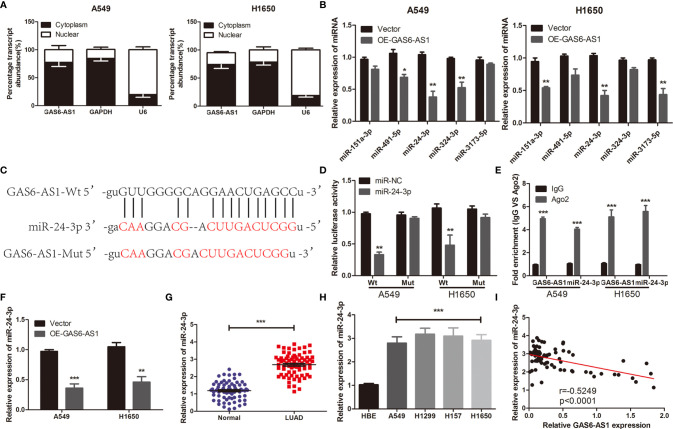
GAS6-AS1 sponges miR-24-3p in LUAD cells. **(A)** GAS6-AS1 is expressed in the cytoplasm of LUAD cells. **(B)** The expression levels of five putative miRNAs in LUAD cells were determined using qRT-PCR. **(C)** The complementary binding of miR-24-3p and wild or mutant type of GAS6-AS1. **(D)** Overexpression of miR-24-3p significantly decreased luciferase activity in the Wt-GAS6-AS1 group. **(E)** The interaction between miR-24-3p and GAS6-AS1 was analyzed in LUAD cells using RIP. **(F)** miR-24-3p expression levels were examined in LUAD cells transfected with Vector or OE-GAS-AS1. **(G)** qRT-PCR analysis of the expression of miR-24-3p in LUAD tissues and normal tissues. **(H)** Relative miR-24-3p expression was measured in four human LUAD cell lines (A549, H1299, H157 and H1650) and HBE. **(I)** The correlation between miR-24-3p and GAS6-SA1 in LUAD was analyzed. Wt, Wild-type; Mut: mutant-type. *P < 0.05, **P < 0.01 and ***P < 0.001.

To clarify the mechanisms underlying the function of GAS6-AS1 in LUAD, the Starbase 3.0 database was used to predict the competitive endogenous RNA (ceRNA) network of GAS6-AS1, which was found to contain five miRNAs and 176 mRNAs (**Figure 3**). The KEGG pathways associated with the target genes are shown in **Additional Figure 2**. Accumulating evidence has shown that cytoplasmic lncRNA may be part of a ceRNA network of miRNAs that negatively regulates the expression of mRNA (20, 21). Therefore, we determined the expression levels of GAS6-AS1 in the nucleus and cytoplasm of LUAD cells. GAS6-AS1 was mainly expressed in the cytoplasm of A549 and H1650 cells, suggesting that GAS6-AS1 might play a negative role in regulating miRNAs (**Figure 4A**). The potential miRNA targets of GAS6-AS1 were determined using StarBase 3.0. QRT-PCR was then used to determine the levels of five miRNAs (miR-151a-3p, miR-491-3p, miR-24-3p, miR-324-3p and miR-3173-5p) that were hypothesized to bind to GAS6-AS1 in LUAD cells (**Figure 4B**). Because miR-24-3p displayed the same changes in cells, it was selected for subsequent experiments. The results suggested that miR-24-3p might bind to GAS6-AS1 (**Figure 4C**). Luciferase activity was used to confirm whether there was a direct interaction between GAS6-AS1 and miR-24-3p. MiR-24-3p transfection significantly decreased luciferase activity in the GAS6-AS1-Wt group, but not in the GAS6-AS1-Mut group, indicating direct binding between miR-24-3p and GAS6-AS1 (**Figure 4D**). Compared with IgG, GSA6-AS1 and miR-24-3p were enriched in miRNA ribonucleoprotein complex (MiRNPs) containing Ago2, which was indicative of GAS6-AS1 binding to miR-24-3p (**Figure 4E**). Additionally, GAS6-AS1 overexpression decreased the levels of miR-24-3p in A549 and H1650 cells. Moreover, miR-24-3p was highly expressed in LUAD tissues and cell lines (**Figures 4G, H**) and negatively correlated with GAS6-AS1 expression (**Figure 4I**). The localization of GAS6-AS1 and miR-24-3p were localized to the cytoplasm of cells (**Additional Figure 3**). In conclusion, MiR-24-3p interacted with GAS6-AS1 and downregulated its expression in LUAD cells.

### GAS6-AS1 Regulates the GIMAP6 Expression *via* miR-24-3p

**Figure 5:**
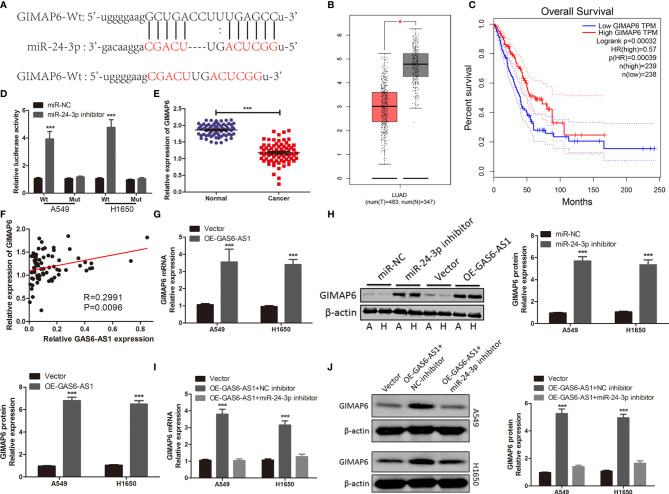
GIMAP6 is a direct target of miR-24-3p in LUAD cells and is positively regulated by GAS6-AS1. **(A)** The complementary binding of sequences of miR-24-3p and wild or mutant type of GAS6-AS1. **(B)** The level of GIMAP6 expression in TCGA database. **(C)** The overall survival associated with GIMAP6 in TCGA database. **(D)** miR-24-3p inhibitor significantly increased luciferase activity in Wt-GIMAP6 group. **(E)** The level of GIMAP6 expression in LUAD samples. **(F)** The correlation between GIMAP6 and GAS6-AS1 in LUAD. **(G, H)** The mRNA and protein levels of GIMAP6 in GAS6-AS1-overexpressing A549 and H1650 cells. **(I, J)** GIMAP6 mRNA and protein expression levels in LUAD cells co-transfected with OE-GAS6-AS1 and either miR-24-3p inhibitor or NC inhibitor. *P < 0.05 and ***P < 0.001.

It is reported that lncRNA regulates expression and stability of mRNA by acting as a miRNA sponge (22, 23). Hypothetical targets of miR-24-3p were predicted using bioinformatics analysis. The 3’-UTR of GIMAP6 contained hypothetical binding sites for miR-24-3p (**Figure 5A**). GIMAP6 was reported to be associated with LUAD (24, 25) and was therefore chosen for subsequent experiments. The expression levels of GIMAP6 in LUAD and its association with survival were determined using the GEPIA database. High GIMAP6 expression levels were associated with good prognosis (**Figures 5B, C**). We carried out a luciferase reporter assay to determine whether the 3’-UTR of GIMAP6 mRNA was directly targeted by miR-24-3p. The inhibition of miR-24-3p expression significantly increased the luciferase activity of GIMAP6-Wt, but not GIMAP6-Mut, indicating that the Mut miR-24-3p binding site was successfully constructed (**Figure 5D**). GIMAP6 expression levels in LUAD samples and their relationship with miR-24-3p expression were also assessed. qRT-PCR demonstrated that the expression of GIMAP6 in LUAD tissues was significantly lower than in normal tissues (**Figure 5E**). Furthermore, there was a negative correlation between GIMAP6 and miR-24-3p expression (**Figure 5F**). In addition, following miR-24-3p inhibition, GIMAP6 mRNA and protein levels in A549 and H1650 cells decreased significantly (**Figures 5G, H**). These findings indicated that miR-24-3p could target GIMAP6 in LUAD cells.

The expression levels of GIMAP6 were measured in A549 and H1650 cells following GAS6-AS1 overexpression, in order to determine whether GAS6-AS1 could regulate GIMAP6 expression. Overexpression of GAS6-AS1 resulted in GIMAP6 upregulation in A549 and H1650 cells, both at the mRNA and protein level (**Figures 5G, H**). LUAD cells were co-transfected with OE-GAS6-AS1 and NC inhibitors or miR-24-3p inhibitors. Following co-transfection with miR-24-3p inhibitor, the expression of GIMAP6 in A549 and H1650 cells overexpressing GAS6-AS1 was downregulated (**Figures 5I, J**). Altogether, these findings suggested that GAS6-AS1 and miR-24-3p were involved in a ceRNA that positively regulated the expression of GIMAP6.

### GAS6-AS1 Overexpression Suppresses LUAD Growth *In Vivo*

**Figure 6:**
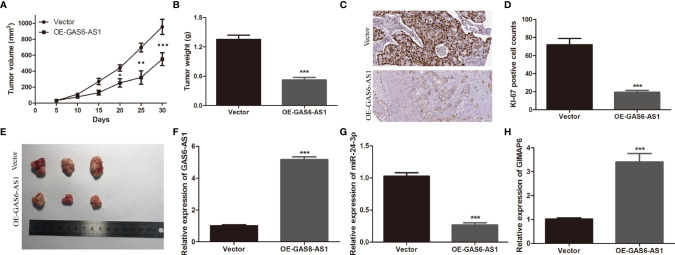
GAS6-AS1 overexpression inhibits LUAD tumor growth *in vivo*. **(A)** The volume of tumor xenografts was measured every 5 days from 1 week after injection. **(B)** Tumors weights in the OE-GAS6-AS1 and Vector groups. **(C)** Tumor weight was measured. **(D, E)** Immunohistochemical staining of Ki-67 in the OE-GAS6-AS1 and Vector groups in xenograft tumor tissues. **(F–H)** qRT-PCR was used to measure the expression levels of GAS6-AS1, miR-24-3p and GIMAP6 in xenograft tissues. IHC, Immunohistochemistry. *P < 0.05, **P < 0.01 and ***P < 0.001.

Tumor xenotransplantation experiments were carried out to determine the effect of GAS6-AS1 *in vivo*. A549 cells transfected with Vector or OE-GAS6-AS1 were injected into nude mice. The tumor volume was measured every five days. It was found that tumor growth was faster in the Vector group than in the OE-GAS6-AS1 group (**Figure 6A**). After 30 days, the tumor tissue was collected from the host. The average tumor weight in the vector group was markedly higher than in the OE-GAS6-AS1 group (**Figures 6B, C**). In addition, Ki-67 staining showed that there were fewer positive cells in the OE-GAS6-AS1 group than in the control group (**Figure 6D**). We then measured the expression levels of GAS6-AS1, miR-24-3p and GIMAP6 by qRT-PCR. Compared with the Vector group, the levels of GAS6-AS1 and GIMAP6 expression in the OE-GAS6-AS1 group were upregulated, while miR-24-3p expression was downregulated (**Figures 6E–H**). These results demonstrated that overexpression of GAS6-AS1 could inhibit tumor progression *in vivo*.

## Discussion

Previous studies have demonstrated that abnormal expression of lncRNA can promote or inhibit the biological function of NSCLC (7, 26). Furthermore, lncRNA is considered to be a potential therapeutic target for the treatment of NSCLC patients (27). Thus, understanding the expression profile and specific functions of cancer-related lncRNA candidates in NSCLC may provide insight into the diagnosis and treatment of NSCLC. In this study, we examined GAS6-AS1 expression levels in LUAD and its functional role and regulatory mechanism in LUAD progression. We found that downregulation of GAS6-AS1 reduced the levels of GIMAP6 by sponging miR-24-3p, which promoted the development of LUAD *in vivo* and *in vitro*.

Several studies have shown that the expression of GAS6-AS1 was dysregulated in many types of cancer, including NSCLC (14–16). Nevertheless, the functional roles and potential mechanism of GAS6-AS1 remain unclear. In this study, we demonstrated that GAS6-AS1 expression was downregulated in LUAD samples and cell lines, in accordance with the results of previous studies (16). In addition, low GAS6-AS1 expression was associated with increased tumor size, clinical stage and lower overall survival rates, indicating that GAS6-AS1 was associated with poor prognosis. Our findings also suggested that the overexpression of GAS6-AS1 could significantly inhibit the proliferation, migration and invasion of LUAD cells. In addition, xenotransplantation experiments indicated that the overexpression of GAS6-AS1 inhibited tumor growth in nude mice. Thus, low expression of GAS6-AS1 played a carcinogenic role in the progression of LUAD.

Accumulating evidence has confirmed that miRNAs regulate the occurrence and progression of various cancer types and can promote or prevent malignant tumors, including NSCLC (28, 29). lncRNA exerts its biological function by targeting miRNA (20, 21). Accordingly, we hypothesized that GAS6-AS1 could affect the occurrence and progression of LUAD through this biological mechanism. Using the online database Starbase3.0 to predict the potential miRNA targets of GAS6-AS1, we identified miR-24-3p as a potential target of GAS6-AS1. Luciferase activity and RIP analysis further indicated that GAS6-AS1 could bind to miR-24-3p in LUAD cell lines. The expression of GAS6-AS1 was negatively correlated with the expression of miR-24-3p in NSCLC samples, and the database also confirmed our conclusion (**Additional Figure 4**). Importantly, the overexpression of GAS6-AS1 downregulated miR-24-3p expression, which inhibited the proliferation of LUAD cell lines. These findings demonstrated that GAS6-AS1 performed its function in LUAD cell lines by sponging miR-24-3p.

Increasing evidence suggests that lncRNA can indirectly regulate target genes expression by binding to miRNA (30, 31). GIMAP6 was the predicted target of miR-24-3p and was expressed at low levels in NSCLC. GIMAP6 is a member of the GTPase immunity-associated proteins (GIMAP) family, which might play a role in the regulation of cell survival (24). Decreased expression of this gene may be associated with NSCLC. Therefore, we further examined the relationship between GAS6-AS1, miR-24-3p and GIMAP6. Overexpression of GAS6-AS1 significantly increased the expression of GIMAP6 and the addition of a miR-24-3p inhibitor attenuated this effect. In addition, GIMAP6 expression levels were positively correlated with GAS6-AS1 in LUAD, which verified the existence of a GAS6-AS1/miR-24-3p/GIMAP6 axis in LUAD. These results indicated that GAS6-AS1 could regulate GIMAP6 and promote the progression of LUAD by targeting miR-24-3p.

In conclusion, this study suggested that GAS6-AS1 was downregulated in LUAD tissues and was associated with poor prognosis in patients with LUAD. Overexpression of GAS6-AS1 inhibited the proliferation of LUAD cells *in vitro* and tumor growth *in vivo* by regulating miR-24-3p and GIMAP6. Altogether, these observations suggested that GAS6-AS1 might represent a potential therapeutic target for LUAD. GAS6-AS1 might still be involved in different mechanisms in NSCLC. In the future, we will increase the sample size to verify our conclusions.

## Data Availability Statement

The original contributions presented in the study are included in the article/**Supplementary Material**. Further inquiries can be directed to the corresponding author.

## Ethics Statement

The studies involving human participants were reviewed and approved by Ethics Committee of Qingdao University. The patients/participants provided their written informed consent to participate in this study. The animal study was reviewed and approved by Animal Ethics Committee of Qingdao University.

## Author Contributions

YW, MM and MW conceived and designed the experiments. YW, MM, CL and YY collected and analyzed data. YW and MW wrote this manuscript. All authors contributed to the article and approved the submitted version.

## Funding

Applied Research Project for postdoctoral researchers in Qingdao supported this study.

## Conflict of Interest

The authors declare that the research was conducted in the absence of any commercial or financial relationships that could be construed as a potential conflict of interest.

## Publisher’s Note

All claims expressed in this article are solely those of the authors and do not necessarily represent those of their affiliated organizations, or those of the publisher, the editors and the reviewers. Any product that may be evaluated in this article, or claim that may be made by its manufacturer, is not guaranteed or endorsed by the publisher.

## Supplementary Material

Supplementary Figure 1 Comparison of GAS6 expression before and after transfection in LUAD cells.

Supplementary Figure 2 The top eight KEGG pathways associated with the GAS6-AS1 ceRNA in LUAD.

Supplementary Figure 3 GAS6-AS1 and miR-24-3p binded in the cytoplasm of cells.

Supplementary Figure 4 The correlation between GAS6-AS1, miR-24-3p and GIMAP6 in LUAD was analyzed using Starbase.
