# Supplementary figures and images for: GAS6-AS1 Overexpression Increases GIMAP6 Expression and Inhibits Lung Adenocarcinoma Progression by Sponging miR-24-3p
Source: Front Oncol. 2021 Aug 26;11:645771. doi: 10.3389/fonc.2021.645771 (PMC8426347; doi:10.3389/fonc.2021.645771)

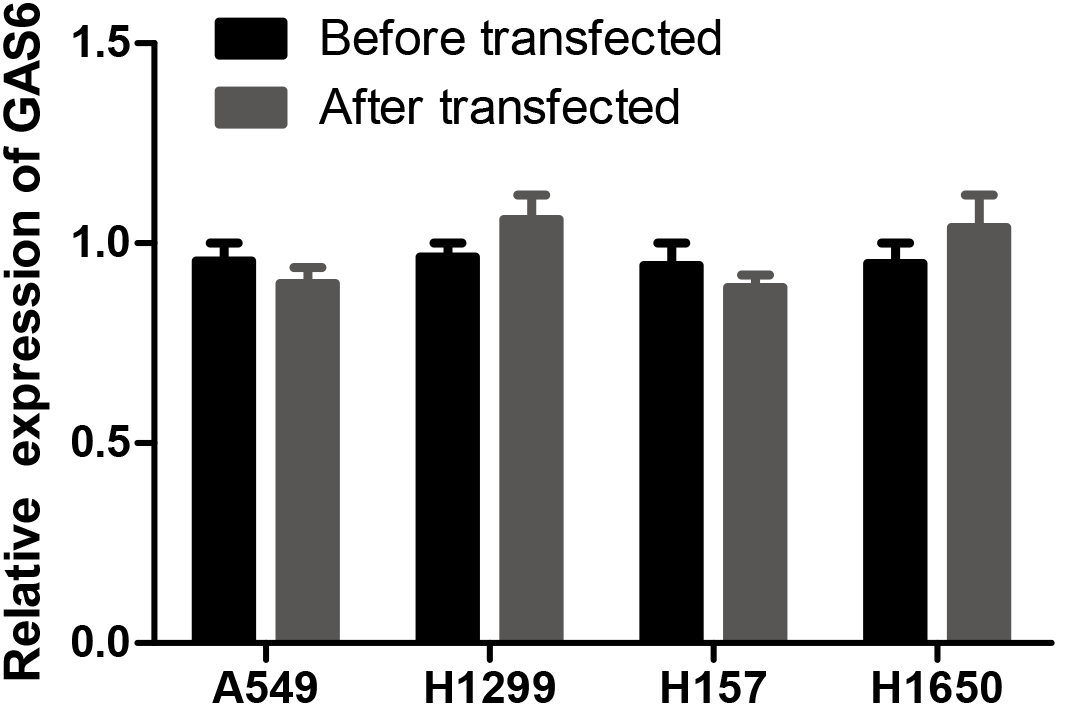

Supplement: Supplementary Figure 1 — Comparison of GAS6 expression before and after transfection in LUAD cells. [file Image_1.tif]

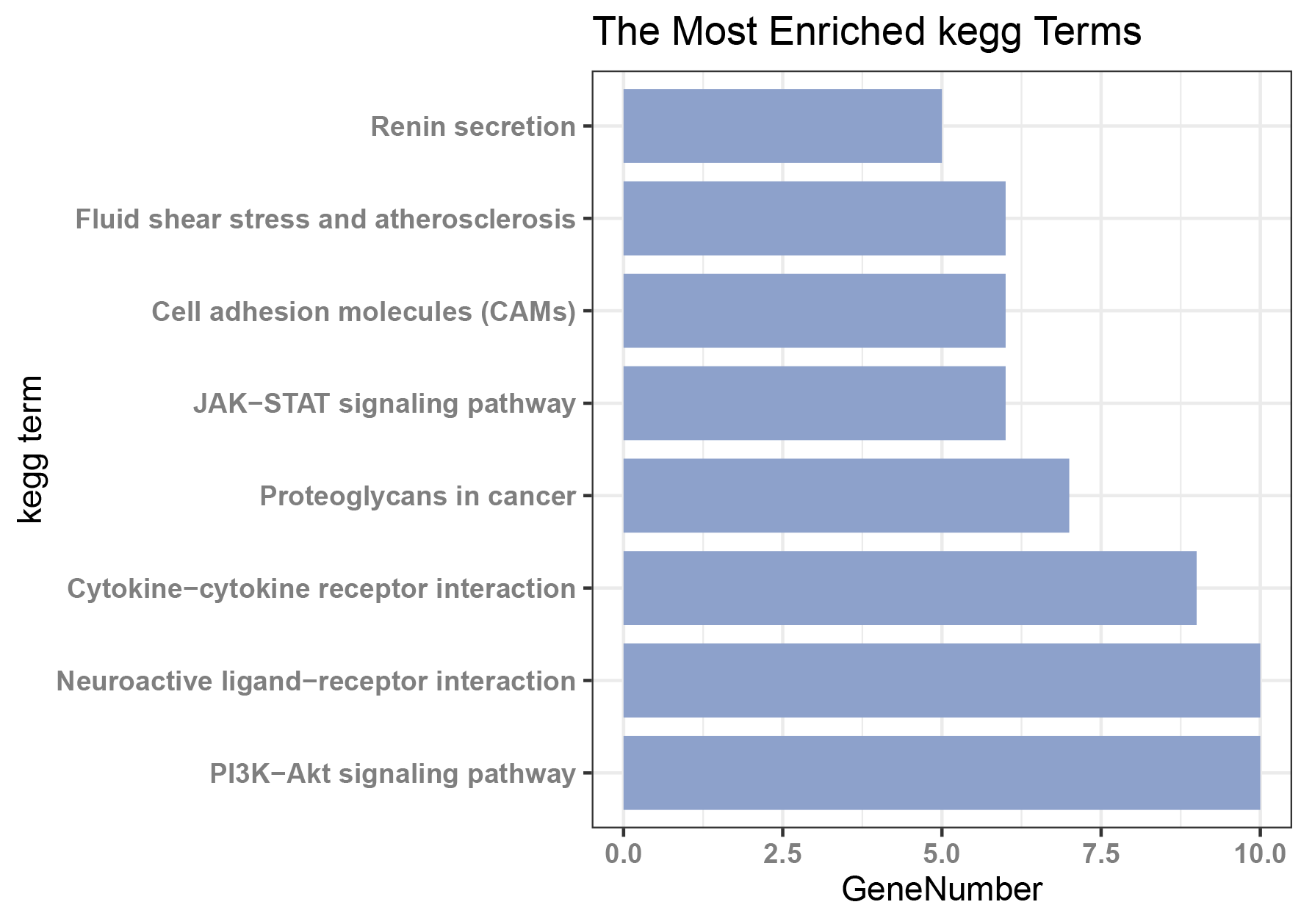

Supplement: Supplementary Figure 2 — The top eight KEGG pathways associated with the GAS6-AS1 ceRNA in LUAD. [file Image_2.tif]

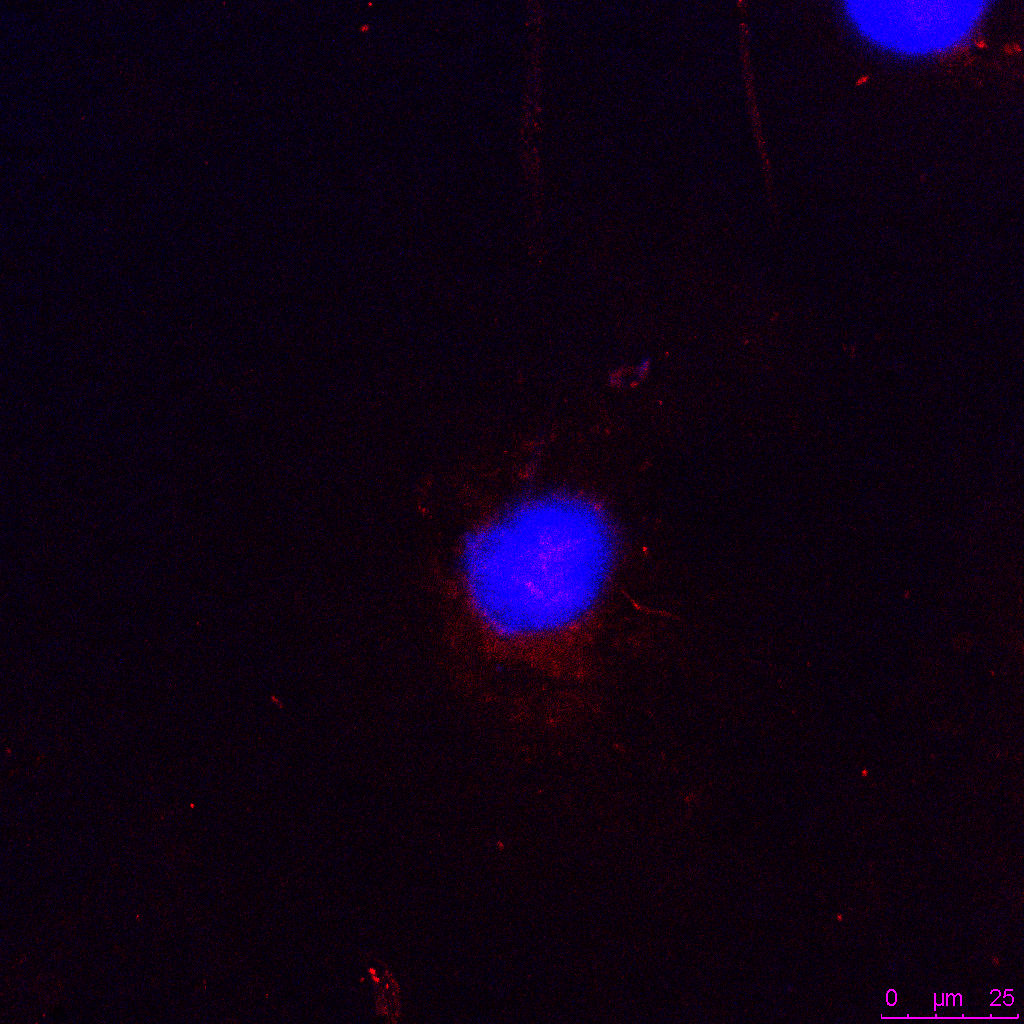

Supplement: Supplementary Figure 3 — GAS6-AS1 and miR-24-3p binded in the cytoplasm of cells. [file Image_3.tif]

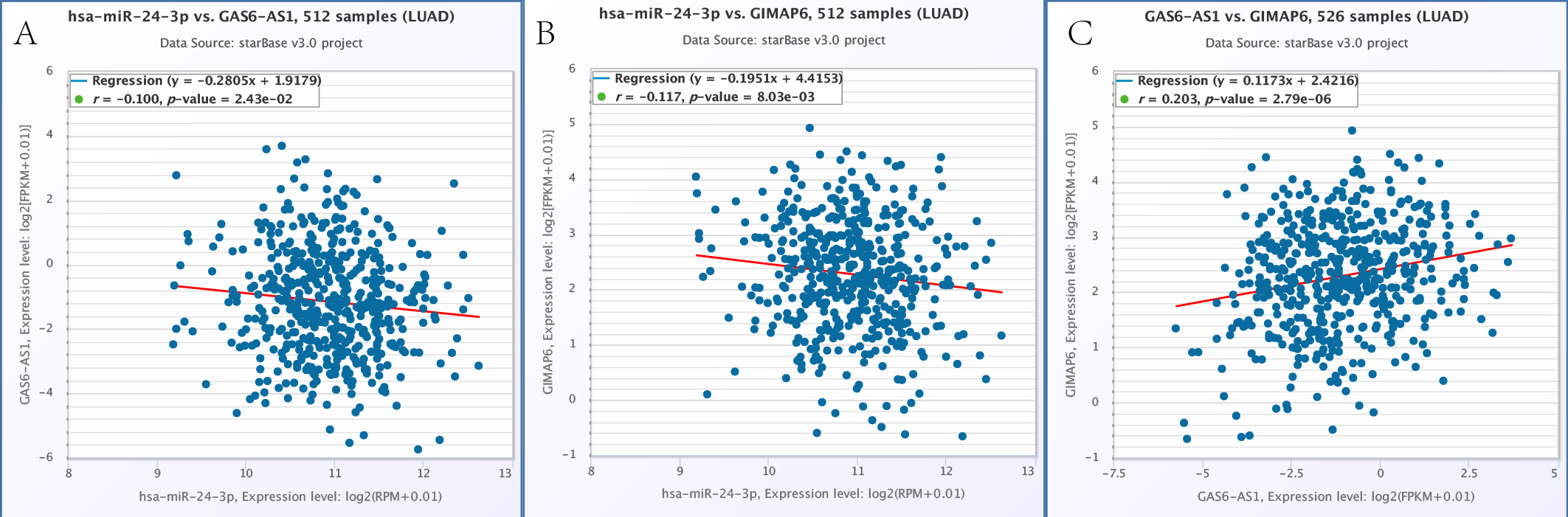

Supplement: Supplementary Figure 4 — The correlation between GAS6-AS1, miR-24-3p and GIMAP6 in LUAD was analyzed using Starbase. [file Image_4.tif]
